# Gatekeeping or Provider Choice for Sustainable Health Systems? A Literature Review on Their Impact on Efficiency, Access, and Quality of Services

**DOI:** 10.3390/jmahp12040029

**Published:** 2024-12-06

**Authors:** Christos Ntais, Nikolaos Kontodimopoulos, Michael A. Talias

**Affiliations:** 1 Healthcare Management Program, School of Economics & Management, Open University of Cyprus, Nicosia 2220, Cyprus; christos.ntais@st.ouc.ac.cy; 2 Department of Economics and Sustainable Development, Harokopio University, 17676 Athens, Greece; nkontodi@hua.gr

**Keywords:** primary healthcare, gatekeeping system, healthcare system efficiency, equity of access, quality of care, patient satisfaction

## Abstract

As early as 1978, the World Health Organization set primary healthcare as the basis on which health systems should be built worldwide. However, the health systems of the different countries show considerable variations in terms of the implementation of gatekeeping from primary to secondary healthcare and direct access to specialists and hospital care. This literature review attempts to present the gatekeeping system with references to the UK, Sweden, the Netherlands, and Germany compared to the situation in Greece, where no gatekeeping system exists. Particular emphasis is placed on the impact of gatekeeping on the healthcare system’s efficiency, equity of access, and the quality of the services provided. Evidence on the effects of gatekeeping is conflicting or limited by the low internal validity. Making the right gatekeeping implementation decisions is difficult in the absence of data. High-quality research studies on health outcomes, clinical efficacy, cost-effectiveness, quality of life, healthcare quality, utilisation of healthcare services, the burden in the healthcare system, and the opinions of patients, physicians, and policymakers are all necessary for developing policy.

## 1. Introduction

Primary healthcare (PHC) serves as the patient’s initial point of contact with a healthcare professional, aiming to diagnose and treat common diseases that do not require specialised attention. A fundamental task of PHC units and services is to direct patient cases requiring more specialised healthcare to dedicated structures. The gatekeeping system is the decision-making process for referring patients to secondary healthcare. It involves the role of primary healthcare physicians or general practitioners (GPs) in authorising access to speciality care, hospital care, and diagnostic tests. This clear delineation of the gatekeeping system’s role in the healthcare system provides a comprehensive understanding of its function and significance.

The need to implement the gatekeeping system arose in response to the welfare state in developed Western countries, characterised by full healthcare coverage of the population, coupled with an increase in life expectancy [1]. In addition, the emergence of cardiovascular, respiratory, and malignant diseases as significant causes of mortality in modern societies, combined with the population explosion, has led to the need for preventive interventions of a long-term nature to meet the health needs of the population without having their hospital structures collapse [2,3,4,5].

In response to all these challenges, PHC is at the core of all efforts in most European countries to improve the health of their population, increase accessibility and quality of healthcare services, eliminate inequalities in providing healthcare services to citizens, and control the cost of these services. The efficiency of healthcare systems adopting the PHC approach is assessed by specific indicators referring to the overall level of health of the population [6], the level of reliability of and equality in accessibility to the healthcare system [7], the quality and cost of services provided [8,9], and the level of patient satisfaction [10].

This review presents the gatekeeping system in the UK (as the country with the longest tradition of gatekeeping), Sweden, the Netherlands, and Germany (as illustrative examples of the three different models of healthcare systems—Beveridge, private, and Bismarck, respectively) and, finally, Greece (as a case study of a complete absence of gatekeeping). Through a critical appraisal of the gatekeeping system, we look at its impact on services efficacy, access, and quality.

## 2. Methods

**Figure 1:**
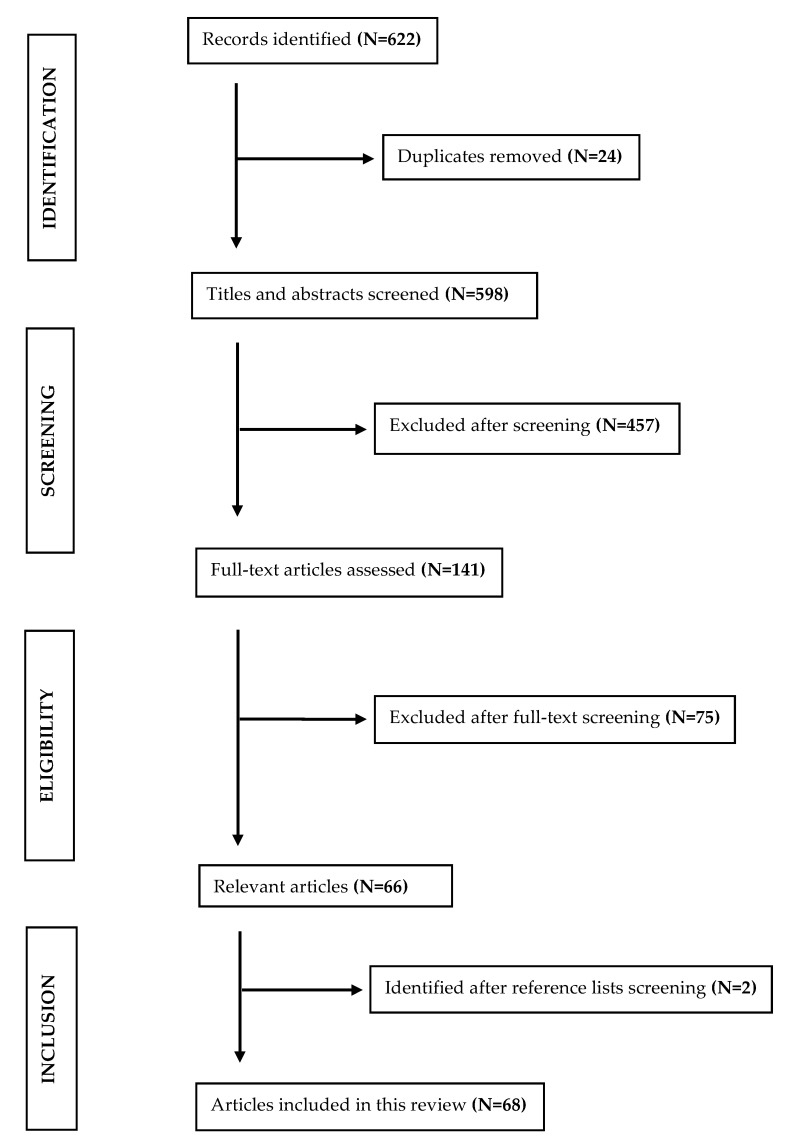
Flow chart of literature selection process.

The method used was a narrative literature review. The relevant literature was searched in the scientific databases PubMed and Scopus up to August 2024, using different combinations of the following terms: “primary healthcare”, “gatekeep *”, “gatekeeping system”, “health * system *”, “health service *”, “efficiency”, “equity”, “access”, “quality”, “patient satisfaction”, “cost”. Articles published in peer-reviewed journals and including primary and/or secondary data, were considered for this review without any chronological and geographical restriction. Studies with no relevance to the gatekeeping system were excluded. Figure 1 illustrates the article selection process for this review.

## 3. Results

### 3.1. The UK PHC System

The British healthcare system falls under the category of national healthcare systems of universal free coverage (the Beveridge model). The Department of Health ensures the provision of healthcare services at the regional level through 10 Strategic Directorates and at the local level through 151 Primary Care Trusts (PCTs) covering about 340,000 inhabitants. PCTs coordinate and deliver PHC through contracting with GPs and professional organisations [11]. PHC relies on GPs, who control the use of healthcare services and the flow of patients to higher levels of healthcare. In other words, GPs function in the PHC system as gatekeepers, i.e., patients are referred to specialised attention and/or hospitals only and strictly through GPs. Individuals can freely choose their GP within the geographical area where they live. However, access to specialists is typically restricted and requires a referral from a GP, except in specific situations such as emergencies [12].

### 3.2. The Swedish PHC System

Sweden has a national healthcare system of universal coverage (the Beveridge model). Insured individuals can choose their preferred healthcare centre and family physician [13]. Disincentives such as higher charges at hospital outpatient clinics and longer waiting times were introduced to address the flow to hospitals. Since 2005, the “0-7-90-90” rule has been in place, i.e., an immediate visit to a PHC provider within the day, a visit within seven days to a GP, a visit within 90 days to a specialist, and finally, a maximum wait of 90 days between diagnosis and treatment [13].

### 3.3. The Dutch PHC System

The Dutch healthcare system belongs to systems with a strong alternative presence of private insurance [14]. In the Netherlands, GPs manage most cases and act as gatekeepers regarding access to specialists and hospitals. Therefore, GPs have a key role in the country’s PHC as they are the first healthcare professional a citizen visits when they present a health issue. Insured individuals can choose a GP, through whom and after a referral, they can consult a specialist. Basic insurance includes an essential package of services, which is the same for everyone, but extra insurance is possible for additional benefits [14].

### 3.4. The German PHC System

The German healthcare system belongs to the category of social insurance (the Bismarck model). In Germany, PHC is controlled by a network of freelance physicians, most often with the speciality of GP or paediatrician, who lead small teams providing healthcare services to citizens. A referral to secondary healthcare can be made directly, but the re-examining and treatment of complications are performed at the PHC level [15]. In essence, Germany does not have a gatekeeping system. However, some incentives are provided to the insured individuals to enrol in family medicine schemes and follow a more efficient gatekeeping system. Each insured individual can choose a GP or specialist from the list of those contracted with their social security fund while visiting hospital outpatient clinics requires certain conditions [15].

### 3.5. The Case of Greece

In most developed Western countries, PHC is considered the first point of contact with healthcare services, usually at a general practice. In Greece, efforts to create an integrated PHC system based on GP/family physician services have been unsuccessful [16]. When the reform of the Greek National Health System (NHS) was halted in 1987, three pathways of organising PHC were created: (a) in non-urban areas, the NHS health centres, staffed by GPs and other healthcare professionals, came into operation (the Beveridge model in the UK); (b) in urban areas, physicians contracted with the various professional social security funds were the first point of contact (the Bismarck model in Germany); (c) the Social Insurance Foundation (“IKA”) had its polyclinics operating where physicians of all specialities were on duty (the Semashko model of the former USSR). In 2011, the creation of the National Organization for the Provision of Healthcare Services (“EOPYY”) consolidated all social security funds and everyone gained the right to go wherever they saw fit. The system became unified but more complex. In 2014, with the transfer of the IKA’s polyclinics from EOPYY to the Primary National Healthcare Network (“PEDY”), the integration of the IKA into the NHS took place. The subordination of PEDY units to the Healthcare Regions (“YPE”) and the healthcare centres consolidated the administrative scheme but did not affect the daily access to healthcare services. In 2017, local healthcare units (“TOMY”) were created to provide family medicine services in urban areas but ended up being just another healthcare service like the others. In conclusion, while the laws passed on PHC describe well-organised services, this was never seen in practice [16]. Implementing the gatekeeping system has been the subject of several healthcare reforms that have attempted to turn PHC physicians into gatekeepers to control the flow of patients within the NHS. The complete failure of this venture is mainly due to the chronic pathologies of the Greek public administration system. The main weaknesses plaguing PHC in Greece are summarised in Table 1.

## 4. Discussion

GPs are the gatekeepers to most healthcare services in several European countries; however, their role in controlling patient flow to specialists is the most contentious issue of gatekeeping [28]. There has been a long-lasting discussion about the clinical, economic, and ethical impacts of gatekeeping [29,30,31,32]. There are good arguments both for and against gatekeeping (Table 2).

In a perfect world, gatekeeping should ensure that patients only consult with specialists for conditions their GP cannot treat and are directed to the appropriate specialised physician thus freeing up specialists’ time for more complex cases. However, the argument that gatekeeping is an effective cost-reduction method may be incorrect [34]. For example, significant differences in the GDP spent on healthcare between countries with and without a gatekeeping system have not been ascertained [33,35,36].

Gatekeeping has been related to late diagnosis and negative outcomes [37,38]. European countries operating robust gatekeeping systems have consistently demonstrated a lower rate of survival for cancer patients [37], although the effect on diagnosis seems varied [39]. A few studies indicate that health impact and quality of life for patients in gatekeeping models might be similar to those in direct access models [40,41].

There is inconsistent and limited evidence on the effect of gatekeeping on the quality of healthcare and satisfaction for patients or providers [40,41]. Patient satisfaction is substantially poorer when policies restrict direct access to specialists, particularly when those policies reject patient requests for referrals (e.g., for a second opinion) [42,43], though not always [44]. These kinds of dissatisfaction are typically linked to poorer treatment adherence and outcomes. By deciding to refer to the GP, gatekeeping undermines the person-centred approach, patient choice, and shared decision-making—elements many governments hope to advance. However, some have argued that gatekeeping could shorten the time patients wait to see specialists, improving patient satisfaction [33].

While the gatekeeping system through GPs plays a crucial role in coordinating patient care and maintaining healthcare system efficiency, GP-requested exams can, in certain scenarios, act as barriers to timely and effective specialist consultations due to logistical, financial, or accessibility issues [45,46,47,48]. Addressing these barriers requires a multifaceted approach, including balanced policies that ensure patients can complete necessary exams promptly thereby reducing delays and optimising the intended benefits of the gatekeeping approach. Balancing the benefits of gatekeeping with the need to minimise delays and burdens on patients is essential for optimising healthcare delivery and patient outcomes [45,46,47,48].

Giving patients immediate access to specialists and expanding their provider options could exacerbate disparities in healthcare use and quality [49]. Private speciality care is used more in states where GPs are gatekeepers [50]. In France, incentives encouraging gatekeeping impede access to specialists, especially for the underprivileged and uninsured individuals covered by complementary insurance [51]. Nonetheless, data from European states indicate that gatekeeping lessens healthcare disparities [49,52,53], helps underprivileged groups make health-related decisions, and reduces the need for advantaged groups to visit specialists unnecessarily [54], as the latter tend to make use of specialist services more frequently [49,53].

As already mentioned, a critical functional parameter of PHC is the ability of its staff to refer cases of patients requiring more specialised healthcare to structures organised for this purpose. The primary purpose of implementing the gatekeeping system is to reduce the operational costs of the healthcare system while maintaining the quality of the services provided. The gatekeeping system can serve this purpose by increasing communication between the different levels of healthcare service provision, reducing unnecessary referrals to secondary healthcare, making more sparing use of specialised healthcare services, and minimising the likelihood of errors [55,56].

In general, implementing the gatekeeping system in PHC is part of the effort to control the cost of healthcare services [57]. A key tool is the control of patient flow within the healthcare system through barriers at the primary level [57]. However, implementing a gatekeeping system does not necessarily imply improving the quality of services [58]. Thus, it should be noted that the effectiveness of the gatekeeping system in controlling healthcare expenditure does not necessarily go hand in hand with high levels of patient satisfaction with the quality of services provided. Indeed, empirical studies have concluded that the constraints imposed by the gatekeeping system create discomfort among citizens and, in some cases, foster antagonistic attitudes between primary and secondary healthcare physicians [59]. In addition, the gatekeeping system has been challenged as a benefit-cutting measure that prevents patients’ free use of the healthcare system and encourages the delegation of medical responsibilities to third parties (e.g., nurses). The gatekeeping system de facto restricts free choice within the system. It promotes the emergence of paramedical specialities that often take over healthcare services at a lower cost [60].

In a systematic review of 25 studies, Sripa et al. [61] investigated the effects of gatekeeping implementation on healthcare quality, health service utilisation and expenditure, health-related outcomes, and patient satisfaction. Gatekeeping was associated with lower health service use and expenditure levels, better quality of care, and more appropriate referrals for further hospital visits and investigations. However, one study reported adverse effects on cancer patients. In particular, the survival rate of cancer patients under gatekeeping was significantly lower than that of patients in direct access systems. However, implementing gatekeeping was not otherwise associated with the delayed referral of patients. Therefore, concerns were raised about the accuracy of diagnoses made by gatekeepers. Implementing gatekeeping led to fewer hospitalisations and the use of specialist care but was inevitably associated with more visits to primary healthcare. Patients were less satisfied with the gatekeeping system compared to the direct access systems.

In another systematic review of 26 studies, Velasco Garrido et al. [40] attempted to evaluate the impact of the gatekeeping system on health levels, health service utilisation, and cost by looking at parameters such as quality of life, patient satisfaction, quality of care, health service utilisation, and economic outcomes (expenditure or efficiency). In most studies, the implementation of gatekeeping was associated with lower levels of health service utilisation (up to −78%) and lower healthcare expenditure (up to −80%). However, there was considerable heterogeneity in the size and direction of the results of individual studies. The researchers concluded that the evidence on the effects of gatekeeping is of limited quality. While there are several studies on the effects of gatekeeping on healthcare services use and expenditure, its effects on health outcomes, patients’ quality of life, quality of care, and the satisfaction of patients and healthcare professionals have only been assessed in studies that do not reach clear conclusions.

A study conducted in Switzerland [62] investigated to what extent the cost savings of gatekeeping compared to a fee-for-service system are due to more efficient resource management or to stratification and risk selection techniques. In particular, healthcare costs between a group of beneficiaries of a gatekeeping system and a group of beneficiaries of a fee-for-service system were compared for the year 2000. The total expenditure per beneficiary in the gatekeeping system group was 8% lower than in the fee-for-service group. The estimated cost savings achieved by replacing the fee-for-service system with gatekeeping in the study population was 15–19% per beneficiary. The researchers concluded that the gatekeeping system achieved significant cost savings not attributable to stratification and risk selection techniques.

Many analysts in the UK claim that the nation’s low healthcare spending, compared to other European countries, is the result of gatekeeping. Forrest [28] notes that while it is true that nations with gatekeeping systems pay less for healthcare than those without similar referral management, gatekeeping is not solely responsible for the lower expenses. Rather, gatekeeping mechanisms have evolved in countries where healthcare resources are scarcer. The supply side controls cause decreased costs rather than demand management at the primary care–speciality care interface [28].

A recent survey of 1234 regular GPs in Norway looked at their role as gatekeepers in protecting the secondary healthcare system during the COVID-19 public health crisis [63]. In the spring of 2020, Norwegian GPs treated and triaged patients suspected of having COVID-19 during the first lockdown. In Norway, most patients with suspected COVID-19 symptoms were treated in primary healthcare during the early stages of the pandemic; just 3.6% of suspected cases were admitted to hospitals, indicating that hospitals were adequately protected. This probably shielded secondary healthcare services from capacity limit breakdown and potentially harmful exposure to contagion.

Finally, Mbau et al. [64] systematically reviewed the efficiency of health systems across various countries, focusing on how health systems transform inputs into desired outcomes. The authors found that efficiency improvements were linked to better healthcare infrastructure, more integrated care systems, and gatekeeping mechanisms in PHC. However, they also pointed out that efficiency gains sometimes came at the expense of equity, especially in lower-income settings where resource access was more limited. The study emphasised the need for tailored efficiency strategies based on specific country contexts and income levels.

## 5. Conclusions

What level of gatekeeping is necessary? How can we manage a sustainable healthcare system while also enabling patient choice? Do we want a healthcare system so clogged with red tape and delays that its users feel it is not working? Achieving the ideal balance is difficult. A well-worked gatekeeping strategy strikes a compromise between patient choice, system limitations, and therapeutic demands. Policymakers might be concerned that if gatekeeping is laxened, many patients will burst through the doors of specialists. However, this may be more of a concern than a fact: in a large, capitated multispecialty group practice, the average number of visits to GPs declined following the shrinkage of a gatekeeping system, but the average number of visits to specialists remained the same, to the exclusion of the visits of chronically ill children to specialists, which increased [65,66]. Other possibilities to consider include giving clinical commissioning groups direct access to some specialist services or giving up the gatekeeper function for specific patient groups. In some circumstances, facilitating more straightforward access to specialists or other medical practitioners may be more cost-effective while yielding therapeutic advantages. For instance, it has been demonstrated that self-selection for individuals with musculoskeletal issues reduces long-term pain and disability, increases patient satisfaction, and reduces waiting times and expenditure [67].

Making the right gatekeeping implementation decisions is difficult in the absence of data. Evidence on the effects of gatekeeping is conflicting or limited by the low internal validity. Estimates of GPs’ referral patterns under popular payment models (such as capitation and fee-for-service) exist [68]. Still, no data exists on the completely or partially relaxed gatekeeping results. Evidence on health outcomes, clinical efficacy, cost-effectiveness, quality of life, healthcare quality, utilisation of healthcare services, the burden in the healthcare system, and the opinions of patients, physicians, and policymakers are all necessary for developing policy. It is important to carefully assess pilot programmes that gradually loosen gatekeeping for particular speciality areas within primary healthcare to ensure the clinical and non-clinical advantages exceed the drawbacks. It is also important to assess the various gatekeeping forms, including co-payment and incentives. We must determine if higher healthcare costs are a natural consequence of easier specialist access. We also need to understand the intended and unintended effects of stringent gatekeeping. In a system that applies integrated care, gatekeeping should be a supplemental mechanism that creates a more lenient division between primary and secondary healthcare, allowing individuals needing specialised care to receive it more promptly.

## Figures and Tables

**Table 1:** Primary healthcare (PHC) deficits in Greece.

PHC Deficits in Greece	Reference(s)
Fragmentation of responsibilities and lack of central planning in the production and distribution of PHC services	[17]
Lack of continuity in healthcare due to the absence of a gatekeeping mechanism and patient referral system	[18]
Human resources are not oriented and trained in PHC services, are focused on healthcare without guidelines, and are unevenly distributed geographically	[19]
Inadequate funding of PHC with serious shortcomings in logistical infrastructure and staffing, and limited availability of services from evening hours onwards	[20]
Inequalities in access due to long waiting times, high costs, and geographical constraints, particularly for vulnerable groups	[21]
Delays in the integration of new technologies	[22,23]
Lack of quality control of services and low efficiency and user satisfaction	[24]
Increased private expenditure due to the involvement of the private sector in healthcare services, informal payments, and induced demand	[25,26]
Absence of interventions and services for the management of chronic diseases, mental disorders, home care, prevention, and health promotion	[27]

**Table 2:** Arguments for and against gatekeeping.

For	Against
Reduced use of healthcare services and lower expenditure	Increased costs due to delayed diagnosis; money saved on specialist access is spent elsewhere in the system
Shorter waiting times to see a specialist	Discourages patients from seeing a specialist if they feel the GP is not resolving their case
Referral mechanisms are needed as the healthcare system cannot support everything patients want	Undermines the importance of patient choice, empowerment, and shared decision-making
Build expertise by ensuring specialists see more complex cases	Lack of clinical knowledge as GPs only treat simple and common cases
Improves patient safety and protects patients from the adverse effects of over-treatment/over-use	May affect clinical outcomes due to delayed diagnosis
Reduces inequalities	Increases inequalities
A referral system increases information flow and communication between GPs and specialists	Maintains the traditional GP–specialist divide, hindering collaboration
Strong gatekeeping arrangements have no negative impact on service satisfaction	Causes conflict in the patient–physician relationship and affects patient satisfaction
GPs are more likely to treat specialist cases and have exposure to a wider range of specialist cases	Increased workload for GPs
Cost containment and system efficiency	Financial considerations can lead to over- or under-referral, and GPs may have underlying interests

Adapted from [33].

## Data Availability

Not applicable.
